# Kidney Disease and Youth Onset Type 2 Diabetes: Considerations for the General Practitioner

**DOI:** 10.1155/2012/237360

**Published:** 2012-01-18

**Authors:** Allison B. Dart, Elizabeth A. Sellers, Heather J. Dean

**Affiliations:** ^1^Department of Pediatrics and Child Health, University of Manitoba, 840 Sherbrook Street, Winnipeg MB, Canada R3A 1S1; ^2^Section of Nephrology, Department of Pediatrics and Child Health, Children's Hospital of Winnipeg, FE009 840 Sherbrook Street, Winnipeg, MB, Canada R3A 1S1

## Abstract

Youth onset type 2 diabetes (T2DM) continues to increase worldwide, concomitant with the rising obesity epidemic. There is evidence to suggest that youth with T2DM are affected by the same comorbidities and complications as adults diagnosed with T2DM. This review highlights specifically the kidney disease associated with youth onset T2DM, which is highly prevalent and associated with a high risk of end-stage kidney disease in early adulthood. A general understanding of this complex disease by primary care providers is critical, so that at-risk individuals are identified and managed early in the course of their disease, such that progression can be modified in this high-risk group of children and adolescents. A review of the pediatric literature will include a focus on the epidemiology, risk factors, pathology, screening, and treatment of kidney disease in youth onset T2DM.

## 1. Epidemiology of Youth Onset Type 2 Diabetes

Type 2 diabetes (T2DM) has been described in children and adolescents since the 1980s [1], and coincident with the rising obesity epidemic, the incidence and prevalence have continued to rise over the last thirty years [2, 3]. Youth onset T2DM has now been described around the world, including Canada, Japan, India, Australia, the United States (US), and the United Kingdom (UK) [4–9]. The highest rates have been reported in the Pima Indian population in the US, with a prevalence of 1.4% in boys and 2.88% in girls between 10 and 14 years [10]. In Canadian First Nation children 4–19 years of age, the prevalence has been reported to be as high as 1% in some communities [11]. In most other populations, although rates are increasing, the disease remains comparably rare. In the US, for example, the incidence in 10–14 years old is 8.1/100,000 person years, and 11.8 per 100,000 person years in children 15–19 years [12]. In Canada, a recent active surveillance initiative revealed a minimum incidence rate of T2DM in children less than 18 years of 1.54 per 100,000 per year. The highest rate was seen in the province of Manitoba, with a minimum incidence rate of 12.45 per 100,000 children [13]. The lowest rates have been reported in the UK at 0.53 per 100,000 in less than 17 year olds [14].

In addition to obesity, multiple other risk factors for the development of youth onset T2DM have been identified. Firstly, most affected children belong to minority ethnic groups including Canadian First Nation, American Indian, Hispanic, African-American, and Indo-Asian [6]. T2DM represents only 6% of non-Hispanic white children in the US with diabetes [15] but accounts for 46.1% of newly diagnosed Hispanics, 57.8% of non-Hispanic blacks, 69.7% of Asian Pacific Islanders, and 86.2% of American Indians with youth onset diabetes [12].

A strong family history is almost universal, with 45–80% of children with T2DM having at least one parent and 70–100% having a first or second-degree relative affected with the disease [16, 17]. The intrauterine environment has also been shown to be important. Children at the lowest and highest extremes of birth weight are at increased risk [18], as are those exposed to pregestational or gestational diabetes in utero [19, 20]. In contrast, breastfeeding has been shown to be protective [19, 20]. Finally, specific genetic factors may play an important role. For example, a unique hepatic nuclear factor- (HNF-)1*α* is a transcription factor expressed in many tissues including the liver, intestine, pancreatic *β*-cell, and kidney. A polymorphism of this gene (HNF-1*α* G319S) has been identified in the Oji-Cree language group of First Nation people in Manitoba and northwestern Ontario. It is associated with an insulin-secretory defect, which predisposes to early onset T2DM in this population [21, 22].

## 2. Nephropathy Associated with Type 2 Diabetes

In adults, T2DM is the leading cause of end-stage kidney disease (ESKD) accounting for 30–40% of cases in most countries. ESKD secondary to diabetic nephropathy typically manifests after 20 to 30 years of diabetes exposure [23]. In children and adolescents, diabetes accounts for only 0.1% of ESKD [24]. However, there is mounting evidence to suggest that renal complications in youth onset T2DM manifest themselves early in the course of disease, and that progression parallels that seen in adult onset T2DM [25]. As youth onset T2DM has only been described for twenty years, we are just now starting to see the impact of the renal complications associated with this devastating disease, as the first cohort of youth enter their third decade.

Microalbuminuria is the first manifestation of a renal complication of diabetes and is the most commonly reported complication of T2DM in youth [26–30]. Reported rates vary widely between 7 and 22% at presentation [27, 28, 31] and between 9.6 and 72% within 3–10 years after diagnosis [26–32]. Variation in rates depends mainly on the definition of albuminuria utilized in each study. Most studies report albuminuria in one random urine sample, which overestimates the prevalence of pathologic albuminuria, as urinary albumin excretion can be transient. Studies that have utilized more stringent criteria (2 out of 3 abnormal samples over a 3–6 month period) report more conservative rates, such as the TODAY study cohort, which reported a prevalence of albuminuria of 13% at a mean age of 14 years, and mean diabetes duration of 7.8 months [33]. This cohort had an average hemoglobin A1c (HbA1c) of 5.9% at enrollment and is therefore likely a low-risk group. In contrast, rates of microalbuminuria in a Manitoba, Canada, cohort with an average HbA1c of  8.9%, based on at least 2 abnormal samples, are much higher at 26.9%, at a mean age of  16.5 years and mean duration of diabetes of 3 years [34]. There has yet to be a study in youth that has reported rates of persistent albuminuria confirmed with a first morning urine sample or overnight urine collection, which are considered the gold standard tests. Nevertheless, these high rates in adolescence are concerning, as microalbuminuria is predictive of progressive diabetic nephropathy, declining glomerular filtration rate (GFR), and cardiovascular disease [35–38].

In the Pima Indian population of the US, there is a 5-fold increased risk of age-specific ESKD in those diagnosed with diabetes before the age of 20 compared to those with diabetes onset between 25 and 54 years of age [39]. However, after controlling for confounders, age at onset was no longer associated with an increased incidence of ESKD, suggesting that the longer duration of diabetes accounted for the increased risk in middle age. These results are in keeping with a previous study by Krakoff et al. which directly compared youth with T2DM <20 years at diagnosis versus young adults 20–39 years versus older adults >40 years at diagnosis which did not show a difference in risk of nephropathy over 25 years between groups [25]. What remains especially concerning, however, is the young age at which youth with T2DM will reach ESKD, requiring dialysis or kidney transplant to sustain life. In Manitoba, ESKD has previously been reported to occur prior to the age of 30 years in young adults diagnosed with T2DM prior 18 years [40]. The same cohort has also recently been shown to have a 4-fold increased risk of ESKD compared to youth with type 1 diabetes (T1DM). In addition, the renal survival fifteen years after diagnosis is 92%, and only 55% in those individuals with 20 years of followup [34]. A higher incidence of nephropathy in young adults with T2DM compared to those with T1DM has also been reported in the Japanese population [41]. A subgroup of this population with proliferative retinopathy prior to age 35 was associated with diabetic nephropathy in 60% and renal failure in 23%, with a requirement for renal replacement therapy at 35 years of age [42].

## 3. Risk Factors for Progression

Kim et al. followed youth with T2DM longitudinally for a median of 3 years, and microalbuminuria identified on one initial urine assessment remained persistent in >93% of youth less than 20 years of age [45]. This study also demonstrated that microalbuminuria in adolescents with T2DM is a predictor of progression to macroalbuminuria over a median followup of 8.1 years [45]. Albuminuria detected in adolescence has also been associated with a 4-fold increased risk of renal failure in early adulthood [34]. Microalbuminuria can therefore be considered a harbinger of renal injury in youth with T2DM, consistent with adult onset T2DM.

The adult literature has identified clinical risk factors associated with the development of diabetic nephropathy. There may also be a genetic predisposition, as has been shown in the Pima Indians in the US [46] as well as in Caucasians [47]. A study of 191 normoalbuminuric adults with T2DM followed prospectively for 5 years described a risk of microalbuminuria of 5% per year and identified male sex, older age, baseline albuminuria, HbA1c, cholesterol, and presence of retinopathy as risk factors [48]. In addition, glomerular hyperfiltration (i.e., increased GFR) has been well described in adults with T2DM, hypothesized to be secondary to concomitant obesity and hyperglycemia. The GFR has been shown to progressively increase and reach a plateau once microalbuminuria develops. Once progression to macroalbuminuria occurs, the GFR begins to decline [49]. It is, however, not consistent in the literature that hyperfiltration is pathogenic, as some studies have shown no association between hyperfiltration and decreased GFR [49].

The Diabetes Control and Complications Trial (DCCT) and the United Kingdom Prospective Diabetes Study (UKPDS) have shown that the intensity of glycemic control significantly impacts the development of diabetic nephropathy in T1DM and adult onset T2DM [50–52]. The pediatric literature is scant but available data suggests that glycemic control is also an important risk factor in youth with T2DM [30, 53]. However, the ideal target for HbA1c to minimize the risk of nephropathy in youth with T2DM has not yet been determined. The clinical practice guidelines currently extrapolate from adult data to target an HbA1c ≤7% [54]. Unfortunately, this target HbA1c is difficult to achieve in youth, due in part to adolescent behavior and nonadherence to treatment recommendations.

Youth with type 2 diabetes have a high prevalence of co-morbidities such as obesity, hypertension, and dyslipidemia [55, 56]. The role of these potentially modifiable clinical risk factors in the development of diabetic nephropathy has not yet been clearly defined in youth onset T2DM. Obesity is associated with glomerular hyperfiltration and the development of glomerulosclerosis and kidney failure [57, 58]. Renal hyperfiltration and hypertrophy may develop in the setting of T2DM in response to disproportionate weight gain and declining insulin sensitivity [59]. According to this hypothesis, adolescents with T2DM may be particularly at risk for premature renal injury relative to adults who experience more gradual weight gain and insulin resistance in adulthood.

Hypertension is highly prevalent in youth with T2DM, with a reported prevalence between 10 and 73% at diagnosis [28, 55, 60–64]. In contrast to the T1DM and adult onset T2DM literature [65], which consistently demonstrates hypertension to be an important modifiable risk factor for the development and progression of diabetic nephropathy, the association between blood pressure control and microalbuminuria in adolescents with T2DM is inconsistent [32, 45, 53]. A small case-control study (*n* = 23) revealed that daytime systolic blood pressure was ~8 mmHg higher among youth with T2DM, relative to normoalbuminuric controls [32]. In contrast, multivariate regression analyses from a larger cross-sectional study and a prospective cohort study demonstrated that systolic blood pressure is not associated with microalbuminuria in adolescents and young adults with T2DM [30, 31, 53]. These different findings may be explained by differences in measurement techniques (casual clinic-based measures versus 24 hr blood pressure monitoring), underpowered studies, or a lack of prospective studies with adequate followup.

Dyslipidemia is a frequent finding in youth with T2DM [27, 28, 31, 55, 62, 64, 66–68]. Two small studies have shown increased LDL cholesterol and triglyceride levels in youth with T2DM and microalbuminuria compared with those with normal albumin excretion [30, 32], suggesting that modification of dyslipidemia may affect risk of nephropathy.

Smoking is reported in 7–48% of youth with diabetes [69, 70]. In T1DM, smoking has been shown to increase the risk of microalbuminuria [71]. In addition, smoking is also associated with a reduced GFR in adults with T1DM and T2DM even after controlling for multiple confounders, including microalbuminuria [72]. Strategies to help with smoking cessation are therefore very important for these high-risk youth.

## 4. Pathology

Classic diabetic nephropathy is characterized by glomerular hypertrophy, basement membrane (GBM) thickening, and mesangial matrix expansion [73]. There is very little biopsy data available on youth with T2DM. In a cohort of ten Canadian First Nation youth with T2DM and macroalbuminuria who underwent renal biopsy, nine of ten biopsies exhibited immune complex disease or glomerulosclerosis, and none had classic diabetic nephropathy [74]. This may in part be due to the high burden of nondiabetic primary renal disease in Canadian First Nation populations [75–77]. Adults with T2DM have also been shown to have nondiabetic glomerular disease, either superimposed on diabetic nephropathy (17%) or more commonly without underlying diabetic disease (28%) [78]. Concomitant obesity is also associated with focal glomerulosclerosis and renal failure [57, 58]. Therefore, there may be early changes seen in youth with T2DM related to nondiabetic kidney disease, and obesity. The additive effects of diabetes and its associated comorbidities may alter progression of renal dysfunction over time.

## 5. Screening

Canadian, American, and International guidelines [15, 54, 79] all recommend screening for diabetic nephropathy at first presentation of diabetes. There are no validated definitions for albuminuria in youth, therefore, the adult values are currently utilized to stage patients (Table 1).

**Figure 1:**
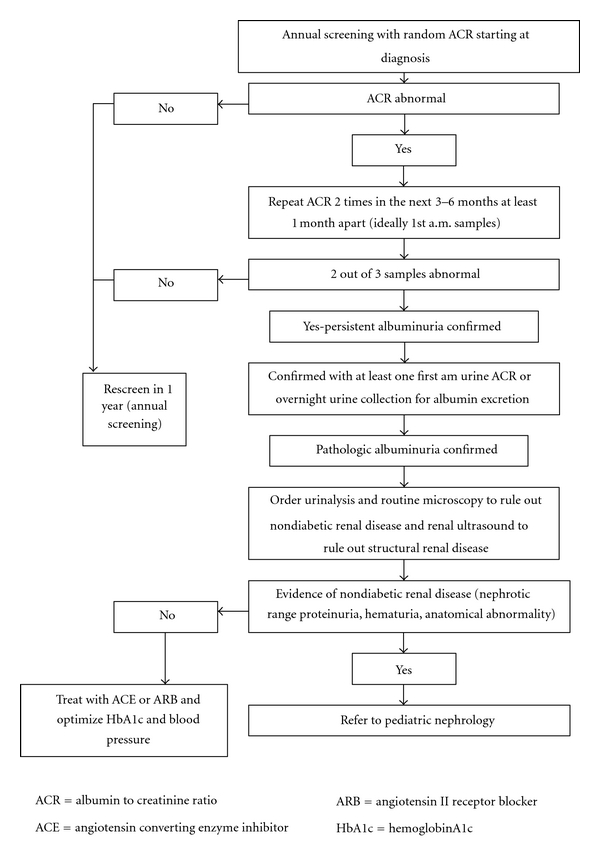
Screening algorithm for albuminuria in youth with type 2 diabetes (modified from CDA guidelines) [43].

Albumin excretion rates in adolescents are influenced by several factors including orthostatic changes, fever, infection, and physical activity. Therefore, it is necessary to have at least 2 positive samples over 3 to 6 months, separated by at least 1 month to confirm the diagnosis [43]. In addition, the diagnosis in youth should be confirmed with a first-morning urine sample or overnight urine collection, to rule or orthostatic proteinuria [80]. An algorithm for screening and treatment has been proposed (Figure 1).

In addition to screening for albuminuria, screening for concomitant comorbidities (dyslipidemia, hypertension, and smoking) is also recommended [54, 79, 81]. An assessment of renal function should also be considered in the form of an estimated glomerular filtration rate [43, 82]. A urinalysis and a renal ultrasound should be performed. The presence of hematuria or red blood cell casts raises the possibility that a nondiabetic kidney disease could be present. In those cases, a glomerulonephritis workup should be initiated, and a renal biopsy considered. In the event of macroalbuminuria, evidence of nondiabetic kidney disease, or an atypical course, a referral to a pediatric nephrologist is recommended.

## 6. Treatment of Nephropathy and Associated Comorbidities (Table 2)

First line in the prevention and treatment of nephropathy associated with T2DM is lifestyle modification and behavior change, including weight reduction, low-sodium diet, and exercise [17] in order to optimize glycemic control and reduce comorbidities such as obesity, hypertension, and hypercholesterolemia. Smoking cessation strategies should be implemented. Unfortunately, this type of therapy requires significant buy in from patients, families, and health care providers. This patient population is particularly challenging to treat due to their adolescent age, as well as the very high rates of lower socioeconomic status (SES). In the TODAY study, 41.5% of participants had a household income <$25,000 [33], and in Manitoba 59.1% of youth with T2DM are in the lowest SES quintile [34]. If lifestyle modification is not sufficient to reduce and maintain HbA1c to <9% (target HbA1c is ≤7%), then pharmacologic management is indicated, in the form of insulin and/or metformin [17, 54, 81], which is the only oral hypoglycemic agent that has been approved (in 2000) by the US Food and Drug Administration [83, 84].

In the absence of concrete blood pressure data in children with diabetes, the current guidelines recommend targeting normal blood pressures in children with T2DM (<90th percentile for age and height, or a maximum of <130/80), [17, 44] not only to potentially reduce the risk of renal injury, but to decrease the risk of cardiovascular disease [17, 54, 81]. Angiotensin II receptor blockers (ARBs) have been used most often in studies of adult onset T2DM and have been shown to consistently reduce the rate of progression from microalbuminuria to macroalbuminura [85–87]. Angiotensin-converting enzyme (ACE) inhibitors have also been evaluated and been shown to decrease the risk of microalbuminuria in normoalbuminuric patients with T2DM [88]. No studies have evaluated these drugs in youth with T2DM. There is a small number of nonrandomized studies in youth with T1DM, all of which have shown reductions in albuminuria with ACE inhibitors [89–91]. Treatment with an ACE or ARB is therefore considered first-line therapy for both hypertension and microalbuminuria in youth with T2DM [17, 81]. If treatment targets are not achieved with one medication, then it is recommended that a second agent be added [17]. Female patients must be advised that congenital malformations, even in the first trimester, have been reported with ACE and ARB use [92]. Contraception counseling is thus very important when these drugs are being used.

Treatment of hyperlipidemia is more controversial. Lifestyle modification is considered first-line therapy, and improvement in glycemic control often results in improved lipid levels. If lifestyle fails, then pharmacologic management with HMG CoA reductase inhibitors is recommended. The Canadian Diabetes Association currently recommends targets used for familial hyperlipidemia for initiation of pharmacologic agents, as studies done in this particular group have not yet been done. A low-density lipoprotein (LDL) threshold value >4.1 mmol/L is utilized to initiate pharmacologic management if there is a family history of early cardiovascular events or ≥4.9 mmol/L in the absence of cardiovascular events [54, 93]. In contrast, the International Society for Pediatric and Adolescent Diabetes recommends a stricter target of <2.6 mmol/L, based on adult data [94].

## 7. Conclusions

The prevalence of type 2 diabetes in youth continues to increase and is associated with microalbuminuria early in the course of disease. The rate of progression to ESKD is in keeping with adult onset disease. Poor metabolic control and co-morbidities such as hypertension, dyslipidemia, and smoking are highly prevalent and likely hasten the progression of diabetic nephropathy and chronic kidney disease. Multifactorial-based prevention and treatment approaches focusing on lifestyle modification and incorporating pharmacologic management of hyperglycemia, hypertension, and albuminuria have been proven in adult studies. Similar strategies are also likely important to delay progression to ESKD in youth with T2DM, however, more research is required to better define the natural history of diabetic nephropathy and optimal treatment targets and therapies in this high-risk population.

## Figures and Tables

**Table 1:** Definitions for albuminuria* [43].

	Albumin : Creatinine ratio (mg/mmol) [43]^¶^	24 hour collection for albumin excretion (mg/day)
Normal	<2.0 (boys)	<30
	<2.8 (girls)	

Microalbuminuria	2.0–20.0 (boys)	30–300
	2.8–28.0 (girls)	

Macroalbuminuria	>20.0 (boys)	>300
	>28.0 (girls)	

^¶^Must be confirmed with either first morning urine sample or overnight urine collection.

*Persistent albuminuria defined as 2/3 positive samples over a 3–6 month period, samples must be at least 1 month apart.

**Table 2:** Recommended treatment targets that may reduce risk of nephropathy in youth with type 2 diabetes.

Clinical parameter	Intervention	Treatment target
Glycemic control	Lifestyle/Insulin/Metformin	HbA1c ≤ 7%
Prehypertension [44] (bp > 90th–95th)	Lifestyle	Bp < 90th percentile
Hypertension [44] (bp > 95th percentile)	Lifestyle ± Ace inhibitor or Angiotensin II Receptor Blocker	Bp < 90th percentile
Dyslipidemia LDL ≥2.6 mmol/L	Lifestyle	LDL < 2.6 mmol/L
Dyslipidemia LDL >4.1 mmol/L	Lifestyle + Statin	LDL < 2.6 mmol/L
Overweight and Obesity	Lifestyle	BMI < 85th percentile
Smoking	Cessation strategies	Nonsmoker
